# Longitudinal trajectories of atherogenic index of plasma and risks of cardiovascular diseases: results from the Korean genome and epidemiology study

**DOI:** 10.1186/s12959-023-00542-y

**Published:** 2023-09-18

**Authors:** Dong-Wook Chun, Yae-Ji Lee, Jun-Hyuk Lee, Ji-Won Lee

**Affiliations:** 1 Department of Family Medicine, Yonsei University College of Medicine, 50-1 Yonsei-ro, Seodaemun-gu, Seoul, 03722 Republic of Korea; 2 Department of Biostatistics and Computing, Yonsei University, Seoul, 03722 Republic of Korea; 3 Department of Family Medicine, Nowon Eulji Medical Center, Eulji University School of Medicine, 68 Hangeulbiseok-ro, Nowon-gu, Seoul, 01830 Republic of Korea; 4 Department of Medicine, Hanyang University School of Medicine, Seoul, 04763 Republic of Korea; 5 Institute for Innovation in Digital Healthcare, Yonsei University, Seoul, 06237 Republic of Korea

**Keywords:** Atherogenic index of plasma, Biomarkers, Cardiovascular disease, Cohort studies, Trajectory

## Abstract

**Background:**

Although the atherogenic index of plasma (AIP) based on a single measurement is a known risk factor for cardiovascular disease (CVD), little is known about whether changes in AIP over time are related to incident CVD. We aimed to determine whether AIP trajectory, which reflects homogenous AIP trends for a particular period, is associated with CVD risk.

**Methods:**

Data from 5,843 participants of the Korean Genome and Epidemiology Study (KoGES) were analyzed. The KoGES had been conducted biennially from the baseline survey (2001–2002) to the eighth follow-up survey (2017–2018). The research design specifies the exposure period from baseline to the third follow-up, designates the latent period at the fourth follow-up, and establishes the event accrual period from the fifth to the eighth follow-up. During the exposure period, we identified two trajectories: a decreasing (*n* = 3,036) and an increasing group (*n* = 2,807) using latent variable mixture modeling. Information on CVD was collected initially through the self-reporting, followed by in depth person-to-person interview conducted by a well-trained examiner. During the event accrual period, the cumulative incidence rates of CVD between the two AIP trajectory groups were estimated using Kaplan–Meier analysis with the log-rank test. Multiple Cox proportional hazard models were used to estimate hazard ratios (HRs) and 95% confidence intervals (CIs).

**Results:**

The increasing AIP trajectory group had a significantly higher cumulative incidence rate of CVD than the decreasing AIP trajectory group. Compared to the decreasing AIP trajectory group, the increasing AIP trajectory group had a higher risk of incident CVD (HR: 1.31, 95% CI: 1.02–1.69) after adjusting for confounders.

**Conclusions:**

The risk of incident CVD increased when the AIP level showed an increasing trend and remained high over a long period. This suggests that checking and managing the trajectory of the AIP can be a preventive strategy for incident CVD.

**Supplementary Information:**

The online version contains supplementary material available at 10.1186/s12959-023-00542-y.

## Background

Cardiovascular disease (CVD) is a global leading cause of mortality [1]. From 1980 to 2017, the global trend for CVD mortality showed an increase by about 21% [2]. In South Korea, CVD has been ranked second among the causes of death between 2009 and 2019, and ischemic heart disease and ischemic stroke ranked sixth and seventh among the top 10 causes of death and disability, respectively [3]. Moreover, there is an increasing trend in the prevalence of CVD as the society ages [1, 4]. As in Europe, the United States, and most developing countries, the socioeconomic cost due to CVD in South Korea is rising and reached 820 million US dollars in 2015 [5, 6].

Risk factors of CVD include modifiable factors such as obesity, hypertension (HTN), diabetes mellitus (DM), dyslipidemia, and cigarette smoking, as well as non-modifiable factors such as sex, age, ethnicity, and heredity [7]. According to the Korean Heart Study that evaluated data from 1997 to 2011, the attributable risk of dyslipidemia for CVD was 8.7% in men and 4.1% in women among modifiable risk factors [8]. As the prevalence of dyslipidemia in Korea increased from 9.0% to 2007 to 20.7% in 2018, the contribution of dyslipidemia to CVD may have increased over time [9]. Among serum lipid profile parameters such as triglyceride (TG), high-density lipoprotein (HDL) cholesterol, and low-density lipoprotein (LDL) cholesterol, serum LDL cholesterol, especially small-dense LDL (sdLDL) cholesterol which is very vulnerable to oxidative damage, has been identified as an atherogenic lipoprotein parameter [10]. However, direct measurements of sdLDL cholesterol levels are limited in clinical practice because of lacking cost-effectiveness and a complicated detection method [11].

The atherogenic index of plasma (AIP) is a simple index based on TG and HDL cholesterol levels [12]. It associates with the lipoprotein particle size and cholesterol esterification rates in apoB-lipoprotein-depleted plasma, and this correlation suggests that the AIP could be an independent biomarker of atherosclerotic diseases [13]. In addition, previous findings have shown that the AIP is inversely proportional to the diameter of LDL cholesterol particles and, thus, reflects the sdLDL cholesterol level [14, 15]. Studies have confirmed the cross-sectional correlation between AIP and CVD risk [16, 17]. However, lipid profiles change continuously throughout a lifetime, as does the AIP, and so far, little is known about the cumulative and serial relationships between AIP trends and cardiovascular outcomes. Thus, it is necessary to determine whether the AIP trend over time is related to the incidence of CVD.

Therefore, this study aimed to determine whether the AIP trajectory, which reflects homogenous AIP trends for a certain period, is associated with CVD incidence using a large-scale community-based cohort dataset.

## Methods

### Study population

The Korean Genome and Epidemiology Study (KoGES) Ansan and Ansung is a community-based cohort study including randomly recruited residents from Ansan, an urbanized city located southwest of Gyeonggi-do, and from Ansung, a rural community south of Gyeonggi-do. For statistical reliability, participants were randomly contacted via home visits, telephone, or mail to recruit representative samples. The baseline survey was performed in 2001–2002, and follow-up examinations were conducted at 2-year intervals until the eighth follow-up (2017–2018). In the present study, we defined the median 5.8 years of baseline to the third follow-up period of the survey as the exposure period (2001–2008), the median 2.1 years of fourth follow-up as the latent period (2009–2010) and the median 7.8 years of fifth to the eighth follow-up period as the event accrual period (2011–2018).

**Fig. 1:**
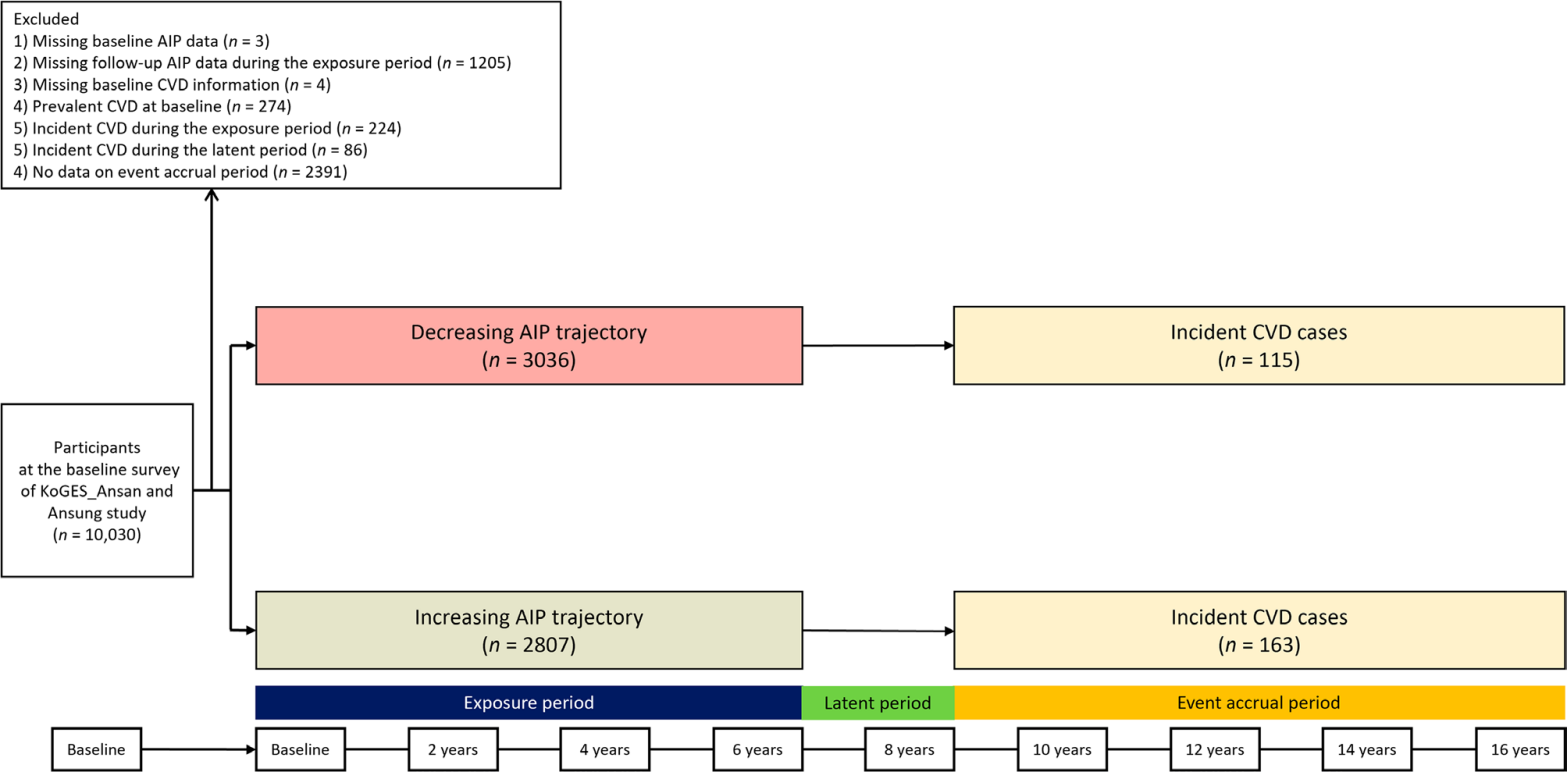
Study flowchart. Abbreviations: AIP, atherogenic index of plasma; CVD, cardiovascular disease

Figure 1 shows the flowchart depicting the study population selection process. Among the participants in the baseline survey (*n* = 10,030), we selected participants without CVD during the exposure period taking the following exclusion criteria into account: (1) missing AIP data at baseline (*n* = 3); (2) missing follow-up AIP data during the exposure period (*n* = 1,205); (3) lack of information about a history of CVD at baseline (*n* = 4); (4) prevalent CVD at baseline (*n* = 274); (5) participants who newly developed CVD during the exposure period (*n* = 224); (6) participants who newly developed CVD during the latent period (*n* = 86), and (7) missing data on the event accrual period (*n* = 2,391). Finally, 5,843 participants were included in the analysis. This study was approved by the institutional review board (IRB) of Nowon Eulji Medical Center (IRB number: EMCS 2022-12-010).

### Assessment of the AIP trajectory groups

The AIP is defined by the following equation [14]:

AIP = log (TG (mg/dL) / HDL cholesterol (mg/dL)).

Using the baseline and follow-up AIP measurements during the exposure period, we performed latent variable mixture modeling to identify trends of changes in AIP. Latent variable mixture modeling and group-based trajectory modeling are representative statistical methods used for trajectory analysis [18]. In our study, we used latent variable mixture modeling to identify AIP trajectories; this approach has the ability to capture potential heterogeneity in AIP trends over time, which might not be fully captured by a predefined set of groups in group-based trajectory modeling [19].

**Fig. 2:**
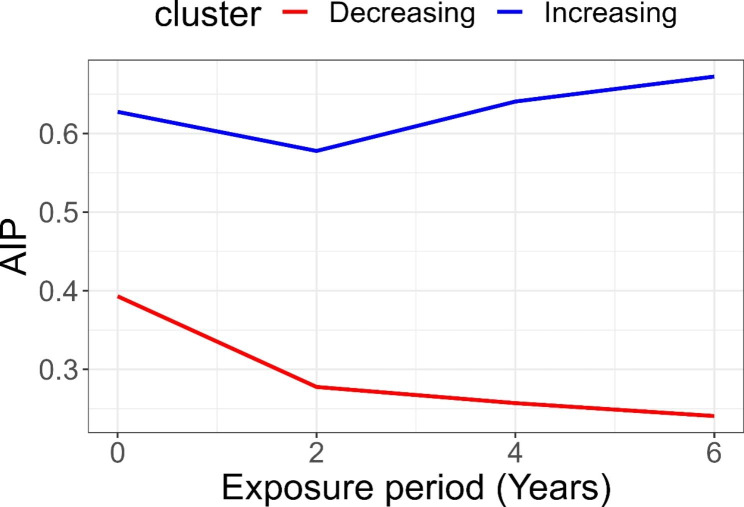
Group-based trajectory modeling according to the atherogenic index of plasma (AIP).

Groups of the study population that maintained relatively homogeneous AIP trends during the study period were classified as AIP trajectories [20–22]. The optimal number of AIP trajectories was identified by applying the minimum Bayesian information criterion (Table S1). Finally, we identified two trajectories: a group with decreasing trend of AIP trajectory (*n* = 3036) and a group with increasing trend of AIP trajectory (*n* = 2807), as shown in Fig. 2, considering the random effect using individual AIP data (Table S2). The AIP ranged between 0.20 and 0.40 in the decreasing AIP trajectory group and between 0.55 and 0.70 in the increasing AIP trajectory group during the exposure period.

### Assessment of CVD

CVD was defined as cerebrovascular accident, peripheral arterial occlusive disease, myocardial infarction, or angina pectoris. CVD history was assessed based on self-reported medical history at each follow-up. If a participant reported an incident CVD event, a well-trained examiner confirmed the case after an in-depth personal interview. In a previous study that utilized data from the same cohort, the concordance between self-reported diagnoses and those ascertained by physician reviews of medical records was 93% [23]. Person-time for each participant was calculated from the start point of the event accrual period (2009–2010) until one of the following were observed: occurrence of CVD, loss to follow-up, or the end of the event accrual period (2017–2018). For individuals who did not experience CVD or were still under follow-up at the end of the event accrual period, person-time was censored at that point.

### Covariates

With reference to the Korean Society for the Study of Obesity, obesity was defined as a body mass index (BMI) ≥ 25 kg/m^2^, which was calculated by dividing weight by the square of height (kg/m^2^) [24]. We measured systolic blood pressure (SBP, mmHg) and diastolic blood pressure (DBP, mmHg) in the sitting position after at least 30 min of rest. The smoking status of participants was classified as never, ex-, intermittent, or daily smoker. The drinking status of participants was classified as non-drinker or current drinker. Regarding the measurement of physical activity, we utilized the International Physical Activity Questionnaire to assess the metabolic equivalents of task (METs)-hours per day (METs-h/day). This was based on participants’ self-reported hours spent in various activities, including sleeping, sedentary behavior (0 METs), very light (1.5 METs), light (3 METs), moderate (5 METs), and heavy (7 METs) physical activities [25]. Participants were subsequently divided into three groups based on their weekly hours of physical activity: low (< 7.5 METs-h/day), moderate (7.5–30 METs-h/day), and high (> 30 METs-h/day) [26]. We calculated the total energy intake (kcal/day) based on a 103-item food frequency questionnaire. Blood samples were collected after at least 8 h of fasting. Fasting plasma glucose (FPG), total cholesterol, TG, HDL cholesterol, C-reactive protein (CRP), and white blood cell counts were analyzed. Serum LDL cholesterol levels were calculated using the Friedewald formula in case of serum TG levels < 400 mg/dL.

DM was defined as glycosylated hemoglobin ≥ 6.5%, FPG ≥ 126 mg/dL, plasma glucose ≥ 200 mg/dL at 2 h in the oral glucose tolerance test, or treatment with anti-diabetic medications or insulin therapy [27]. HTN was defined as SBP ≥ 140 mm Hg, DBP ≥ 90 mm Hg, or treatment with antihypertensive agents according to the 7th Joint National Committee [28]. Dyslipidemia was defined based on any one of the following criteria: (1) serum total cholesterol ≥ 240 mg/dL, (2) serum TG ≥ 200 mg/dL, (3) serum LDL cholesterol ≥ 160 mg/dL, (4) serum HDL cholesterol < 40 mg/dL for men or < 50 mg/dL for women, or (5) treatment with lipid-lowering medications [29].

### Statistical analysis

Continuous variables, including age, BMI, waist circumference (WC), MBP, FPG, serum total cholesterol, TG, HDL cholesterol, LDL cholesterol, CRP, and total energy intake, were expressed as mean ± standard deviation. Categorical variables, including sex, smoking status, current drinking, physical activity, HTN, DM, and dyslipidemia, were expressed as numbers (percentages). Significant differences between continuous variables were assessed using Student’s t-test, whereas the chi-square test was used to analyze differences between categorical variables.

The cumulative incidence rates of CVD between the two AIP trajectory groups were estimated using Kaplan–Meier analysis and compared using the log-rank test. Multiple Cox proportional hazard regression analyses were performed to estimate the hazard ratios (HRs) and 95% confidence intervals (CIs) for incident CVD in the two AIP trajectory groups.

All statistical analyses were conducted using the SPSS software version 23 for Windows (IBM Corp., Armonk, NY, USA) and R software (version 4.1.3; R Foundation for Statistical Computing, Vienna, Austria). We used the R package ‘lcmm’ for latent variable mixture modeling. Statistical significance was set at *P* < 0.05.

## Results

### Baseline characteristics of the study population

The baseline characteristics of the study population are summarized in Table 1. The proportion of men was 46.4%, and the average age was 51.5 years in the total study population. The mean values of BMI, WC, MBP, FPG, total cholesterol level, LDL level and TG level, as well as the proportions of daily smokers, current drinkers, participants with low intensity of physical activity, patients with HTN, patients with DM, and patients with dyslipidemia, were higher in the increasing AIP trajectory group than in the decreasing group. By contrast, the mean values of serum HDL levels were lower in the increasing AIP trajectory group than in the decreasing group.

**Table 1:** Baseline characteristics of the study population

		AIP trajectory groups		
	Total (*n* = 5843)	Decreasing (*n* = 3036)	Increasing (*n* = 2807)	*P* value^*^
Male sex, n (%)	2709 (46.4%)	1225 (40.3%)	1484 (52.9%)	< 0.001
Age, years	51.5 ± 8.5	51.2 ± 8.5	51.9 ± 8.4	0.001
BMI, kg/m^2^	24.6 ± 3.1	24.0 ± 3.1	25.3 ± 2.9	< 0.001
WC, cm	82.5 ± 8.7	80.3 ± 8.8	85.0 ± 7.9	< 0.001
MBP, mmHg	96.2 ± 12.9	94.3 ± 12.8	98.2 ± 12.8	< 0.001
Smoking status, n (%)				< 0.001
Never smoker	3628 (62.9%)	2050 (68.5%)	1578 (56.8%)	
Ex-smoker	875 (15.2%)	415 (13.9%)	460 (16.6%)	
Intermittent smoker	123 (2.1%)	66 (2.2%)	57 (2.1%)	
Daily smoker	1145 (19.8%)	462 (15.4%)	683 (24.6%)	
Current drinker, n (%)	2781 (48.0%)	1362 (45.2%)	1419 (50.9%)	< 0.001
Physical activity, n (%)				< 0.001
Low	413 (7.3%)	211 (7.2%)	202 (7.4%)	
Moderate	3418 (60.7%)	1806 (61.8%)	1612 (59.4%)	
High	1804 (32.0%)	906 (31.0%)	898 (33.1%)	
FPG, mg/dL	86.5 ± 19.6	83.9 ± 14.8	89.4 ± 23.4	< 0.001
Total cholesterol, mg/dL	190.5 ± 34.5	185.6 ± 32.5	195.8 ± 35.8	< 0.001
Triglyceride, mg/dL	160.0 ± 103.1	126.6 ± 62.8	196.1 ± 123.9	< 0.001
HDL cholesterol, mg/dL	44.7 ± 9.9	47.8 ± 10.2	41.3 ± 8.4	< 0.001
LDL cholesterol, mg/dL	115.1 ± 30.9	112.8 ± 29.4	117.7 ± 32.3	< 0.001
CRP, mg/dL	0.2 ± 0.5	0.2 ± 0.4	0.3 ± 0.7	< 0.001
Total energy intake, kcal/day	1968.0 ± 700.6	1973.0 ± 721.1	1962.6 ± 677.8	< 0.001
HTN, n (%)	2130 (36.5%)	942 (31.0%)	1188 (42.3%)	< 0.001
DM, n (%)	656 (11.2%)	225 (7.4%)	431 (15.4%)	< 0.001
Dyslipidemia, n (%)	2755 (47.2%)	946 (31.2%)	1809 (64.4%)	< 0.001

Abbreviations: AIP, atherogenic index of plasma; BMI, body mass index; CRP, C-reactive protein; DM; diabetes mellitus; FPG, fasting plasma glucose; HDL, high-density lipoprotein; HTN, hypertension; LDL, low-density lipoprotein; MBP, mean blood pressure; WC, waist circumference

^*^P value for the comparison of the baseline characteristics between participants of the decreasing AIP trajectory group and those of the increasing AIP trajectory group. Significance was set at *P* value < 0.05

### Incidence of CVD by AIP trajectory group

**Fig. 3:**
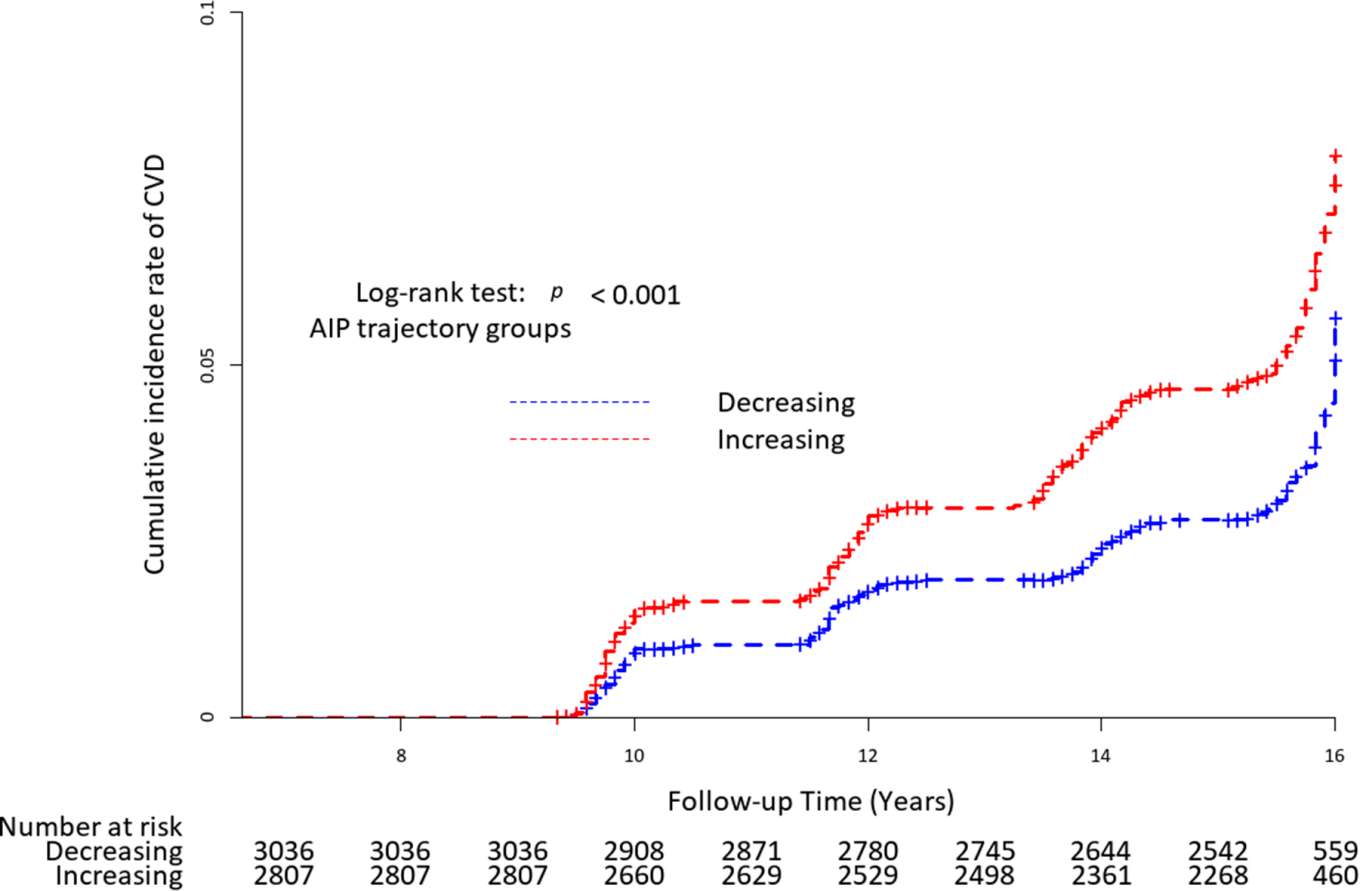
Kaplan–Meier curves of the cumulative incidence of CVD according to AIP trajectory groups. Abbreviations: AIP, atherogenic index of plasma; CVD, cardiovascular disease

During the median 7.8 years of event accrual period, 278 (4.8%) newly developed CVD cases were registered in total. The incidence rate per 2 years ranged from 1.22 to 1.53 (Table 2). Figure 3 presents the Kaplan–Meier estimates of the cumulative incidence rate of CVD by AIP trajectory group. The increasing AIP trajectory group had a higher cumulative incidence rate of CVD than the decreasing AIP trajectory group (*P* for log-rank test < 0.001).

**Table 2:** Incidence of cardiovascular disease during the follow-up

	Exposure period	Latent period	Event accrual period			
Follow-up	Baseline to 3rd f/u	4th f/u	5th f/u	6th f/u	7th f/u	8th f/u
Year range	2001–2008	2009–2010	2011–2012	2013–2014	2015–2016	2017–2018
Total, n	5843	5843	5426	5017	5133	4983
Incidence cases, n			76	61	65	76
Incidence rate per 2 years			1.40	1.22	1.27	1.53

Abbreviation: f/u, follow-up

### Associations of AIP trajectories and incident CVD

Table 3 presents the HRs with 95% CIs for incident CVD in participants with an increasing trajectory of AIP versus those with a decreasing trajectory of AIP. Compared to the decreasing AIP trajectory group, the increasing AIP trajectory group had a higher risk of incident CVD (HR: 1.57, 95% CI: 1.47–2.00). Similar trends were observed in the adjusted models. The adjusted HRs (95% CIs) for incident CVD of the increasing AIP trajectory group were 1.43 (1.12–1.83) in model 1, 1.34 (1.04–1.73) in model 2, and 1.31 (1.02–1.69) in model 3, respectively, compared to those of the decreasing AIP trajectory group.

**Table 3:** Cox proportional hazard regression analysis for incident cardiovascular disease based on the atherogenic index of plasma trajectory groups

	AIP trajectory groups		
	Decreasing	Increasing	
Number, n	3036	2807	
Incident CVD case, n	115	163	
Follow-up period, person-year	22055.2	20101.3	
Incidence rate per 1000 person-year	5.21	8.11	
	HR	HR (95% CI)	*P* value
Incident CVD risk			
Unadjusted	1 (reference)	1.57 (1.47–2.00)	< 0.001
Model 1	1 (reference)	1.43 (1.12–1.83)	0.004
Model 2	1 (reference)	1.34 (1.04–1.73)	0.022
Model 3	1 (reference)	1.31 (1.02–1.69)	0.037

Model 1: adjusted for age, sex, and body mass index

Model 2: adjusted for variables included in model 1 plus smoking status, drinking status, physical activity, and total energy intake

Model 3: adjusted for variables included in model 2 plus hypertension, diabetes mellitus, and serum C-reactive protein

Abbreviations: AIP, atherogenic index of plasma, CVD, cardiovascular disease; CI, confidence interval; HR, hazard ratio

## Discussion

This study aimed to explore the possible relationship between the AIP trajectory and CVD using a large-scale community-based cohort dataset. While a single measurement of AIP can provide valuable information about an individual’s current CVD risk, tracking AIP trajectories over time allows for the identification of individuals who consistently exhibit high AIP levels. Two patterns of AIP trajectories were identified during the exposure period: increasing values and decreasing values. The incident CVD rate in the increasing AIP trajectory group was significantly higher than that in the decreasing AIP trajectory group. Moreover, the increasing AIP trajectory group had a higher risk of incident CVD than the decreasing AIP trajectory group in both crude and adjusted models. Additional analysis by CVD subtypes further indicated their individual significance in relation to AIP trajectories (Fig. S2). This supports the argument that maintaining a high AIP for a long period significantly increases the probability of CVD occurrence.

Considering the contribution of dyslipidemia to the development of atherosclerosis, it is very important to monitor the changes in lipid profiles, especially serum sdLDL levels, since sdLDL induces atherosclerotic lesions [30]. Several studies indicate that through the AIP, considerable information about the lipoprotein spectrum can be inferred, especially about the LDL cholesterol particle size [14–16, 31]. The formula for calculating the AIP incorporates the serum TG and HDL cholesterol levels, and using the AIP as a substitute marker for sdLDL has the advantages of cost-effectiveness and simplicity of its measurement.

While previous studies only determined the correlation between AIP at baseline and incident CVD [32–34], we verified the association of AIP trajectories with incident CVD. Considering that trajectory modeling is a useful statistical method for predicting different outcomes between subgroups of individuals exhibiting similar patterns over time [35], our results, obtained using AIP trajectory modeling, provide valuable insights into the long-term effects of lipid profiles on CVD risk. This analysis also supports the importance of monitoring lipid changes over time and subsequently extends evidence from previous studies [32–34].

Although the mechanisms for the relation of AIP trajectories with CVD risk remain unclear, several possible explanations may account for this relationship. First, increasing AIP trajectory could reflect the increasing trend of insulin resistance. Many studies have investigated the association between insulin resistance and CVD [36–38], and it has been found that hypertriglyceridemia and decreased levels of HDL cholesterol affect insulin resistance [39]. Hypertriglyceridemia inhibits glucose entry into cells and negatively affects glucose oxidation, resulting in insulin resistance [40]. In addition, as the TG level increases, the number and activity of insulin receptors in fat cells decrease [40]. The effect of HDL cholesterol on insulin sensitivity is due to its capability to facilitate glucose uptake into target cells such as hepatocytes, myocytes, and adipocytes [41]. Due to this dyslipidemia-insulin resistance interaction, the TG/HDL cholesterol ratio has been used as an insulin resistance marker [42]. Second, AIP is significantly correlated with the number of very LDL and sdLDL particles, which can upregulate the expression of adherence molecules on endothelial cells [43]. Direct associations between AIP and atherosclerosis have been identified in several studies, including arterial stiffness [44], carotid artery intima-media thickness, and overall state of atherosclerosis [45, 46]. Several studies have demonstrated that the AIP has potential efficacy in predicting CVD risks [13, 16, 31]. In line with these studies, the prevalence of DM, HTN, and incidence of CVD was significantly higher in the increasing AIP group than in the decreasing AIP group. Finally, systemic inflammation is considered a key factor in the development of CVD [47], and inflammatory responses may have mediated the association between AIP and CVD [31, 48]. Zhan et al. [49] suggested that the AIP is a reliable biomarker of ischemic heart disease and reported the relationship between AIP increases and the high incidence of ischemic heart disease mediated by lipid-driven inflammation. Thus, we performed a mediation analysis to determine the effect of serum CRP levels as mediators of CVD development (Table S3). Although CRP is the most commonly used biomarkers of systemic inflammation [50–52], contrary to our expectations, the mediation effect of CRP was not significant. Possible explanations for these results are that pro-inflammatory cytokines other than CRP, such as tumor necrosis factor alpha, interleukin (IL)-1, or IL-6, may act as mediators, and other effects that may cause chronic inflammation could not be completely ruled out in this study. Moreover, genetic differences such as single nucleotide polymorphisms between increasing and decreasing AIP trajectory groups need to be further analyzed. Therefore, further research is needed to identify possible mediators between AIP trajectories and CVD.

This study has several strengths compared to previous studies. First, unlike previous studies, our study confirmed the trend based on trajectories, not spot-checks, which means the study period is the result of data for up to 16 years, including 6 years of exposure, 2 years of latent period and 8 years of event accrual period. To the best of our knowledge, this study is the first to verify that identifying the trend of time-varying variables using trajectory modeling is an important part of CVD prevention. Second, it also has the strength of research using a large population cohort, which demonstrated a robust association between AIP and future CVD risk.

In addition, our research has some limitations. First, selection bias may have occurred because CVD that occurred during the exposure period was not included in the analysis. Second, the prevalence of CVD in the study participants was not directly assessed using the 10th revision of the International Statistical Classification of Diseases code; instead, it was determined through interviews. In this process, recall bias or incomplete reporting may have occurred. Nevertheless, we believe the reliability of this study considering the high concordance rate (93%) of diagnoses of disease between self-reported diagnoses and those ascertained by physician review of medical records [23]. Third, information on mortality was not available. Therefore, the possibility of under-diagnosis of CVD in this study exists. Moreover, it is possible that only healthier people were selected, and the selection bias had rather increased. Fourth, we assumed that changes in AIP during the latent period did not affect CVD events. Fifth, only Korean individuals were included in the current analysis. Considering that the mean value of AIP varies depending on ethnicity [53], our results cannot be generalized to other ethnic populations. Nonetheless, the results of this study still have clinical significance in that AIP trajectories can be a reliable predictor of CVD risk in Asian ethnicities.

## Conclusions

In conclusion, the risk of incident CVD increased when an increasing trend of AIP was maintained for a long time. This suggests that serial monitoring of AIP can be a preventive strategy for incident CVD. Further laboratory research and large-scale clinical studies should be conducted to explain the exact mechanism of the relationship between trajectories of AIP and CVD risk.

### Electronic supplementary material

Below is the link to the electronic supplementary material.

Supplementary Material 1

## Data Availability

The dataset used in this study can be provided after a KCDA review and evaluation of the research plan (https://www.nih.go.kr/ko/main/contents.do?menuNo=300563).

## Abbreviations

CVD: Cardiovascular Disease
AIP: Atherogenic Index of Plasma
KoGES: Korean Genome and Epidemiology Study
TG: Triglycerides
HDL-C: High-Density Lipoprotein Cholesterol
LDL-C: Low-Density Lipoprotein Cholesterol
BMI: Body Mass Index
WC: Waist Circumference
MBP: Mean Blood Pressure
FPG: Fasting Plasma Glucose
CRP: C-Reactive Protein
WBC: White Blood Cell
DM: Diabetes Mellitus
HTN: Hypertension
METs: Metabolic Equivalents of Task
HR: Hazard Ratio
CI: Confidence Interval
