# Supplementary material for: Longitudinal trajectories of atherogenic index of plasma and risks of cardiovascular diseases: results from the Korean genome and epidemiology study
Source: Thromb J. 2023 Sep 18;21:99. doi: 10.1186/s12959-023-00542-y (PMC10506251; doi:10.1186/s12959-023-00542-y)
Supplement: Supplementary file 1 — Supplementary Material 1 [file 12959_2023_542_MOESM1_ESM.docx]

**Supplementary Material**

**Table S1.** Estimation process for the most optimal number of AIP trajectories.

| Number of groups | BIC | Number (percentage) of groups | | | | |
| --- | --- | --- | --- | --- | --- | --- |
|  |  | Group 1 | Group 2 | Group 3 | Group 4 | Group 5 |
| 2 | -4159.1 | 3036 (52.0%) | 2807 (48.0%) |  |  |  |
| 3 | -4254.4 | 0 (0.0%) | 4981 (85.2%) | 862 (14.8%) |  |  |
| 4 | -4228.4 | 0 (0.0%) | 4683 (80.1%) | 0 (0.0%) | 1160 (19.9%) |  |
| 5 | -4202-4 | 0 (0.0%) | 4387 (75.1%) | 0 (0.0%) | 0 (0.0%) | 1456 (24.9%) |

Abbreviations: BIC, Bayesian information criterion; AIP, atherogenic index of plasma.

**Table S2.** Mixed model considering random effect using individual data of AIP.

|  | Estimate | SE | P-value |
| --- | --- | --- | --- |
| Total | -0.016 | 0.001 | <0.001 |
| Decreasing AIP trajectory group | -0.049 | 0.001 | <0.001 |
| Increasing AIP trajectory group | 0.020 | 0.002 | <0.001 |

Abbreviations: AIP, atherogenic index of plasma; SE, standard error

**Table S3.** Mediation effect of inflammatory marker between AIP trajectories and CVD.

| Exposure | Outcome | Mediator | NDE | NIE | TCE |
| --- | --- | --- | --- | --- | --- |
|  |  |  | Estimate (95%CI) | Estimate (95%CI) | Estimate (95%CI) |
| AIP  trajectory group | CVD  occurrence | CRP | 1.56 (1.20-2.02) | 1.01 (0.99-1.03) | 1.58 (1.22-2.04) |
|  | CVD free duration  (Baseline to 9^th^ f/u) |  | 1.58 (1.23-2.04) | 1.01 (0.99-1.03) | 1.60 (1.25-2.06) |
|  | CVD free duration  (5^th^ to 9^th^ f/u) |  | 1.57 (1.22-2.02) | 1.01 (0.99-1.03) | 1.59 (1.24-2.04) |

Abbreviations: AIP, atherogenic index of plasma; CVD, cardiovascular disease; CRP, C-reactive protein; NDE, natural direct effect; NIE, natural indirect effect; TCE, total causal effect.


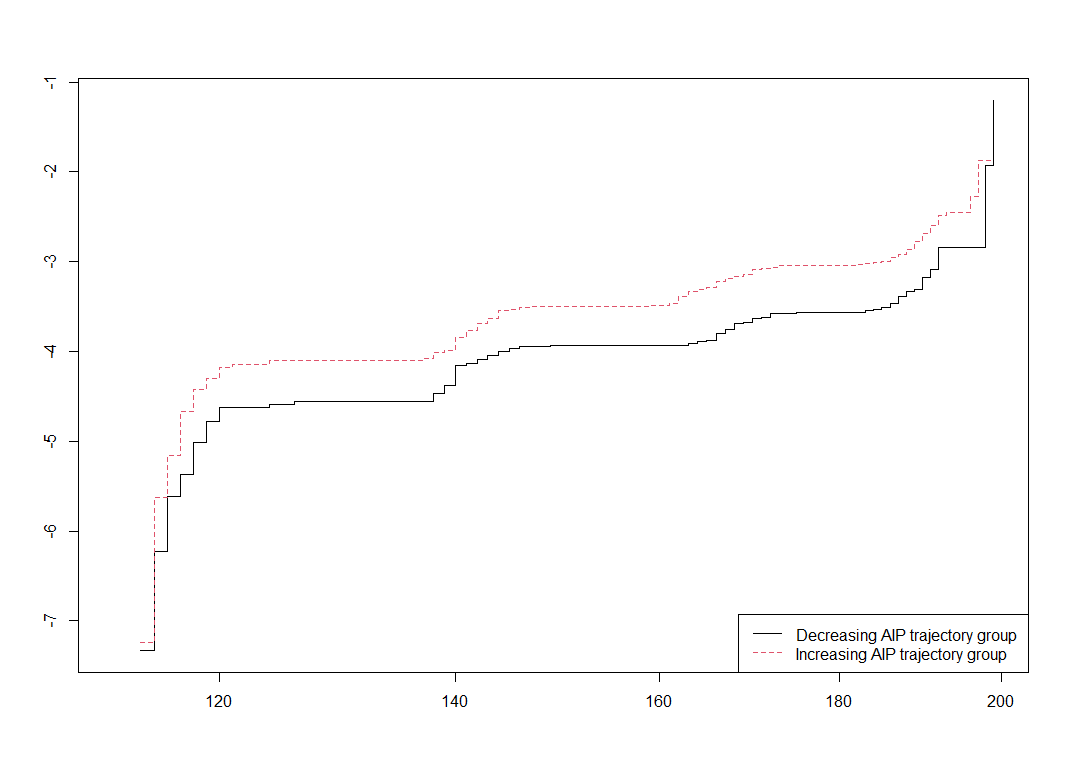


**Figure S1.** Log-log plot for proportional hazards assumption.

Abbreviations: AIP, atherogenic index of plasma


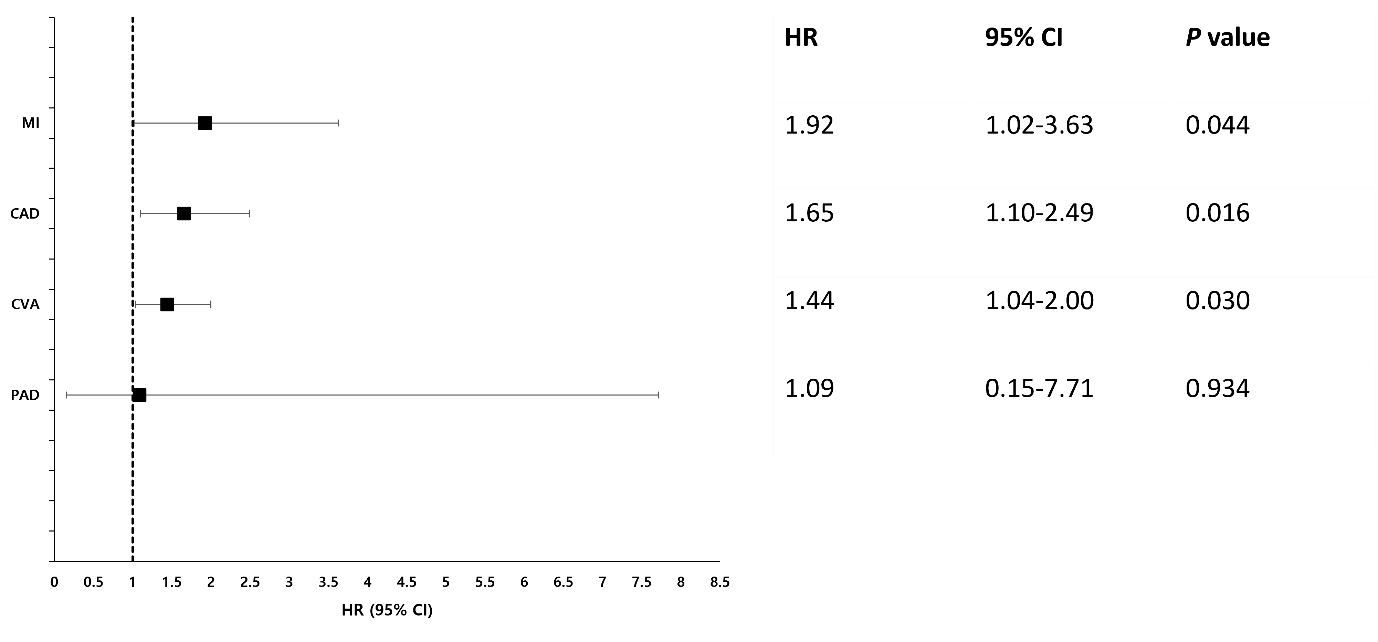


**Figure S2.** Forest plots of CVD subtypes.

Abbreviations: MI, myocardial infarction; CAD, coronary artery disease; CVA, cerebrovascular accident; PAD, peripheral arterial occlusive disease; HR, Hazard Ratio; CI, Confidence Interval.
